# Spinal Posture, Mobility, and Muscle Endurance in Women With Tension‐Type Headache: A Case‐Control Study

**DOI:** 10.1155/prm/5577357

**Published:** 2026-01-12

**Authors:** Mesut Arslan, Sonay Guruhan, Seyda Toprak Celenay

**Affiliations:** ^1^ Department of Physiotherapy and Rehabilitation, Faculty of Health Sciences, Bitlis Eren University, Bitlis, Turkey, bitliseren.edu.tr; ^2^ Department of Physiotherapy and Rehabilitation, Faculty of Health Sciences, Ankara Yıldırım Beyazıt University, Ankara, Turkey, ybu.edu.tr

**Keywords:** mobility, muscle endurance, posture, tension-type headache

## Abstract

**Background:**

Tension‐type headache (TTH), a prevalent form of primary headache, has been linked to alterations in spinal biomechanics.

**Objective:**

This study aimed to compare spinal posture, mobility, and muscle endurance between women with and without TTH.

**Methods:**

This case‐control study included 68 women aged 18–55 years with (*n* = 34, age = 20.51 ± 1.73 years, BMI = 22.03 ± 2.90 kg/m^2^) and without (*n* = 34, age = 19.91 ± 0.99 years, BMI = 21.38 ± 2.99 kg/ m^2^) TTH. Posture (craniovertebral angle) and mobility (range of motion) of the cervical region were evaluated with a goniometer and posture and mobility of the thoracic and lumbar and sacral regions in the sagittal plane were evaluated with the Spinal Mouse device (IDIAG M360, Fehraltorf, Switzerland), cervical region muscle endurance was evaluated with cervical flexion and extension endurance tests and the craniocervical flexion test, and trunk muscle endurance was evaluated with McGill’s trunk muscle endurance tests and the Sahrmann test.

**Results:**

Cervical flexion (95% CI: −1.38 to −0.40, *d* = 0.90, *p* ≤ 0.001) and extension (95% CI: −2.02 to −0.97, *d* = 1.50, *p* ≤ 0.001) mobility and cervical flexor (95% CI: −1.49 to −0.49, *d* = 0.99, *p* ≤ 0.001) and deep neck flexor muscle endurance (95% CI: −1.64 to −0.61, *d* = 1.13, *p* ≤ 0.001) scores were lower in women with TTH compared to the healthy controls. It was also observed that the thoracic angle (95% CI: 0.11 to 1.08, *d* = 0.60, *p* = 0.015) was higher in the sagittal plane and trunk flexor (95% CI: −1.33 to −0.35, *d* = 0.84, *p* = 0.001) and trunk right/left lateral flexor muscle endurance (95% CI: −1.75 to −0.73, *d* = 1.24, *p* ≤ 0.001)/(95% CI: −1.76 to −0.73, *d* = 1.25, *p* ≤ 0.001) and trunk core stability (*p* = 0.003) scores were lower.

**Conclusion:**

Cervical mobility was less and cervical muscle endurance was lower in women with TTH, but their cervical postures were similar. It was also observed that thoracic angle was higher in sagittal plane and trunk muscle endurance was lower.

## 1. Introduction

Tension‐type headache (TTH), the most prevalent form of primary headache, typically presents as mild to moderate bilateral pain with a pressing or tightening quality, unaffected by physical exertion [1]. It has been reported that TTH cases in individuals aged 15–39 years increased by 37% in 2019 compared to 1990 figures and are more common in women [2]. TTH is categorized into two subtypes: episodic (fewer than 15 headache days monthly) and chronic (15 or more headache days per month) [3]. Increased pericranial myofascial tenderness and lower pain threshold and increased myofascial trigger points have been reported in individuals with TTH [4, 5]. The transition from episodic to chronic TTH involves more than just increased frequency; it is marked by central sensitization, greater pericranial tenderness, and a higher comorbidity burden [6]. These factors likely contribute to a higher prevalence and severity of cervical musculoskeletal impairments in chronic TTH [7]. However, the exact etiology and pathophysiology remain unclear. TTH can impair daily activities, reduce quality of life, and lead to work disability [1].

Headache disorders involve modifications in central sensitization processes, particularly within the trigeminocervical pathway, along with dysfunction in pain inhibitory systems [6]. These central changes can be modulated by nociceptive signals originating from peripheral tissues. Thus, nociceptive signals originating from peripheral tissues, such as the spine, muscles, and fascia, could contribute to the triggering of headache episodes [7].

Additionally, the role of the cervical spine in headache disorders is supported by the neuroanatomical convergence of trigeminal and upper cervical afferent pathways within the trigeminocervical nucleus caudalis [8]. This convergence allows for nociceptive input from cervical structures to be perceived as head pain and can also sensitize this pathway, potentially contributing to the pathophysiology of other primary headaches, including TTH. Furthermore, according to the motor adaptation to pain theory, individuals suffering from pain may adopt protective motor strategies. This theory, which can be applied to various pain conditions, suggests that such adaptations are not necessarily pathology‐specific but rather a generalized response. For instance, individuals with headaches—regardless of the primary driver—may exhibit altered motor control and restricted mobility in regions like the thoracic and lumbar spine as a compensatory mechanism to minimize perceived threat or load on the painful area. Indeed, a study in individuals with cervicogenic headaches (CGH) demonstrated this principle, showing reduced postural adaptability throughout the spine compared to healthy controls [9]. While CGH and TTH have distinct diagnostic criteria, this finding illustrates a potential commonality in how different headache disorders might manifest in altered spinal motor control, providing a rationale for investigating similar adaptations in TTH.

Several studies have investigated the relationship between TTH and cervical spine posture, mobility, and muscle endurance [7, 10, 11]. A meta‐analysis demonstrated that individuals with TTH exhibit increased forward head posture and restricted cervical rotation compared to asymptomatic individuals. However, no consistent changes or conflicting results were found regarding muscle performance and motor control [7]. Another study revealed that TTH patients show reduced motor performance due to altered motor control of neck muscles compared to the healthy controls [10]. A separate investigation similarly identified greater forward head positioning and reduced neck mobility among TTH sufferers compared to pain‐free controls [11]. However, only one study has investigated thoracic, lumbar, and sacral spine posture, mobility, and muscle endurance in this population. This study reported a higher lumbopelvic angle in chronic TTH patients compared to the healthy controls [12].

Therefore, this study aims to compare spinal posture, mobility, and muscle endurance between women with and without TTH.

## 2. Materials and Methods

### 2.1. Study Design and Participants

This research employed a case‐control design involving female subjects meeting the International Classification of Headache Disorders (ICHD‐3) diagnostic criteria for TTH. All participant assessments were conducted at Bitlis Eren University’s physiotherapy laboratory during an 8‐month period from June 2024 through February 2025.

The required sample size was calculated a priori using G∗Power software (version 3.0.10, Franz Faul, Universität Kiel, Germany) for an independent samples *t*‐test. To ensure adequate power for a key parameter expected to show a substantial difference, the calculation was based on cervical flexion range of motion. Based on the effect sizes for this parameter reported in previous literature on TTH, which indicated substantial differences between groups, a large effect size (Cohen’s *d* = 0.8) was assumed for the sample size calculation. With an effect size of 0.8, *α* = 0.05 (type I error), *β* = 0.10 (type II error), and a power of 90%, the calculation yielded a minimum total sample size of 68 participants (34 per group) to detect a significant difference between the groups [13].

Inclusion and exclusion criteria: the study enrolled female participants aged 18–55 years, with the TTH group comprising individuals diagnosed according to ICHD‐3 criteria. The TTH group included participants with both episodic (< 15 headache days/month) and chronic (≥ 15 headache days/month) TTH, as per ICHD‐3 frequency criteria [3]. The control group consisted of age‐matched healthy women without headache history. Healthy controls were recruited from patients’ relatives. Participants in the TTH group were not restricted from using physician‐prescribed medications (e.g., painkillers); use of these medications was recorded.

Exclusion criteria encompassed (1) significant comorbidities including neurological (e.g., multiple sclerosis and Parkinson’s disease), rheumatological, cardiovascular, or psychiatric disorders; (2) prior head or spinal surgeries; (3) spinal deformities such as scoliosis; (4) history of malignancy; (5) current pregnancy status; (6) having received any physiotherapy, interventional treatment, or regular manual therapy for headache or neck/back pain in the preceding 1 year; (7) the presence of any other primary/secondary headache disorder according to ICHD‐3 criteria; and (8) cognitive impairments that might compromise study compliance [9].

Healthy control participants were required to have no history of primary or secondary headache disorders (based on ICHD‐3 criteria) and no neck or back pain requiring treatment or limiting activities in the past year. They were also screened using the same exclusion criteria applied to the TTH group.

### 2.2. Data Collection Tools

All assessments were performed by two physiotherapists. Furthermore, to ensure consistency, each parameter was assessed by the same physiotherapist for all subjects.

Demographic characteristics including age, body mass index (BMI), tobacco/alcohol use, and current medications were documented using a standardized sociodemographic form. Physical activity levels were assessed using the short‐form international physical activity questionnaire (IPAQ‐7), which quantifies activity by recording the time (in minutes) and frequency (days) spent on walking and moderate‐intensity and vigorous‐intensity activities (lasting ≥ 10 min) during the last 7 days. The total weekly physical activity volume was calculated as MET‐minutes/week by multiplying the metabolic equivalent (MET) value for each activity category (3.3 METs for walking, 4.0 METs for moderate‐intensity activities, and 8.0 METs for vigorous‐intensity activities) by its duration and weekly frequency. Participants were categorized into three activity levels: high (vigorous‐intensity activity on ≥ 3 days achieving ≥ 1500 MET‐minutes/week or ≥ 7 days of any combination of activities achieving ≥ 3000 MET‐minutes/week), moderate (≥ 3 days of vigorous‐intensity activity of ≥ 20 min/day or ≥ 5 days of moderate‐intensity activity or walking of ≥ 30 min/day or ≥ 5 days of any combination of activities achieving ≥ 600 MET‐minutes/week), or low (not meeting the criteria for “moderate” or “high” categories) [14, 15].

Headache profiles were characterized by documenting onset timing, monthly frequency (in days), episode duration, and pain severity using the numeric pain rating scale (NPRS). The NPRS is an 11‐point scale ranging from 0 to 10, where “0” indicates “no pain” and “10” represents “unbearable pain” [16].

### 2.3. Assessment of Spinal Posture and Mobility

#### 2.3.1. Cervical Posture (Craniovertebral Angle)

The forward head posture was assessed by measuring the craniovertebral angle (CVA) in degrees (°) using a modified universal goniometer. Participants stood in a relaxed standing position looking straight ahead. The goniometer’s axis was positioned over the tragus of the ear, the fixed arm was aligned parallel to the floor, and the moving arm was aligned toward the spinous process of the C7 vertebra. A smaller CVA indicates a greater degree of forward head posture. Inter‐rater reliability assessed by the intraclass correlation coefficient (ICC) was reported to be good (ICC = 0.89) [17].

#### 2.3.2. Cervical Mobility (Flexion and Extension Range of Motion)

Active cervical flexion and extension ROM were measured in degrees (°) using a standard 10‐inch manual goniometer according to standard protocols. For flexion/extension, the goniometer axis was placed over the external auditory meatus, with the fixed arm perpendicular to the ground and the moving arm aligned with the base of the nares. All measurements were performed three times, and the average value was used for analysis. Intraexaminer reliability analysis revealed moderate consistency for goniometric measurements of cervical flexion (ICC = 0.46) and extension (ICC = 0.54) [18].

#### 2.3.3. Thoracic, Lumbar, and Sacral Posture and Mobility

Posture and mobility of the thoracic, lumbar, and sacral regions in the sagittal plane were assessed using the validated, noninvasive, computer‐assisted Spinal Mouse device (IDIAG M360, Fehraltorf, Switzerland). The device was rolled along the midline of the spine from the spinous process of C7 to the S3 during upright standing, maximum flexion, and maximum extension. Posture and mobility measurements in the sagittal plane for thoracic, lumbar, and sacral regions were recorded in degrees (°). Interexaminer reliability was good, with ICCs ranging from 0.62 to 0.93 (mean = 0.81) on day 1 and 0.70–0.94 (mean = 0.86) on day 2, despite minor systematic differences in mean values. For all parameters, intraexaminer reliability was good. ICC values ranged from 0.67 to 0.92 for Examiner 1 and 0.57 to 0.95 for Examiner 2, with the majority (68%) exceeding 0.80. The mean ICC across all parameters was 0.82 for Examiner 1 and 0.84 for Examiner 2 [19].

### 2.4. Assessment of Spinal Muscle Endurance

#### 2.4.1. Cervical Flexor Endurance Test

The cervical flexor endurance test was performed in the supine hook‐lying position. Participants were asked to bring their chin as close to their chest as possible and then lift their head 2.5 cm upward and maintain this position. Intrarater reliability was good to excellent (ICC = 0.82–0.91) and inter‐rater reliability was fair to good (ICC = 0.67–0.78) in the group without neck pain. In the group with neck pain, inter‐rater reliability was moderate (ICC = 0.67) [20]. The duration for which participants could maintain the correct position was recorded in seconds.

#### 2.4.2. Cervical Extensor Endurance Test

For cervical extensor endurance evaluation, subjects lay prone on the plinth with their head extending beyond the edge (initially examiner‐supported). The protocol involved (1) upper thoracic stabilization using a T6‐level strap, (2) application of a weighted (2 kg) occipital headband with integrated inclinometer, and (3) sustained neutral head positioning until failure criteria were reached (weight touching ground or > 5° postural deviation). Intrarater reliability for the neck extensor endurance test was good (ICC = 0.88) [21]. The duration for which participants could maintain the correct position was recorded in seconds.

#### 2.4.3. Craniocervical Flexion Test

The craniocervical flexion test evaluates deep neck flexor muscle endurance. For the test, the participant lies supine in hook‐lying position with arms by the sides and head in neutral position. Prior to measurement, participants were taught to perform craniocervical flexion by gently bringing the chin toward the chest without using superficial cervical flexor muscles. Subsequently, the stabilizer device (Chattanooga, TN, USA) was placed under the suboccipital region (inflated to a 20 mmHg pressure), and participants were asked to press maximally against the device with a “yes” motion and hold for 10 s. The score was calculated by multiplying the amount of pressure increase from baseline by the number of repetitions at that pressure level and was recorded. This digital imaging technique demonstrated excellent reliability, with an inter‐rater ICC of 0.994 and intrarater ICCs ranging from 0.988 to 0.998 for angular measurements [22, 23].

#### 2.4.4. McGill’s Trunk Muscle Endurance Tests

The endurance capacity of the trunk musculature was assessed using McGill’s tests for trunk flexion, extension, and lateral flexion (right and left). Each test measures the maximum hold time (in seconds, s) that an individual can maintain a standardized prone, supine, or side‐bridge position. The tests demonstrated high reliability, with ICCs exceeding 0.97 for assessments conducted over 5 consecutive days and at an 8‐week follow‐up [24].

#### 2.4.5. Sahrmann Test

For the Sahrmann core stability test, while the participant laid supine, the inflatable pad of the pressure biofeedback unit (Chattanooga, TN, USA) was placed in the natural lumbar lordotic curve and inflated to 40 mmHg. Participants were taught the abdominal bracing maneuver. They were then asked to perform different lower extremity movements while maintaining abdominal bracing. The test consisted of 5 stages. The test was terminated when the pressure value on the stabilizer changed by more than 10 mmHg, indicating that the participant could not complete that level. The Sahrmann test showed moderate inter‐rater reliability (ICC = 0.649) [25, 26].

### 2.5. Ethical Considerations

This investigation complied with the ethical principles outlined in the Declaration of Helsinki and adhered to Good Clinical Practice standards. The Institutional Review Board at Bitlis Eren University granted ethical approval for the study protocol (Reference No. 2024/3‐2, April 4, 2024). All participants provided written informed consent prior to study enrollment.

### 2.6. Statistical Analysis

Descriptive statistics included frequency counts, proportional percentages, arithmetic means with standard deviations, and value ranges. Distribution normality was verified using Shapiro–Wilk testing. Parametric analysis with independent samples *t*‐tests compared continuous variables between groups, while chi‐square tests analyzed categorical variables. All analyses were conducted in IBM SPSS Statistics 21.0 (Armonk, NY) and Microsoft Excel 2007. All tests were two‐tailed, and a *p*‐value of < 0.05 was considered statistically significant. Cohen’s convention interprets effect sizes as small (*d* = 0.2), medium (*d* = 0.5), and large (*d* = 0.8) [27].

## 3. Results

The study analyzed data from 34 participants with TTH and 34 age‐matched controls. The data collection process was completed without any dropouts from the initially recruited participants. Comparative analysis of demographic characteristics including age, BMI, smoking/alcohol consumption, and physical activity levels revealed no statistically significant intergroup differences (*p* > 0.05) (Table 1).

**Table 1:** Descriptive data of the groups.

Descriptive parameters	TTH group (*N* = 34)	Control group (*N* = 34)	*p*
Age (years, *X* ± SD)	20.51 ± 1.73	19.91 ± 0.99	0.084^a^
BMI (kg/m^2^, *X* ± SD)	22.03 ± 2.90	21.38 ± 2.99	0.359^a^
Smoking (*n*, %)			
Yes	4 (11.8)	7 (20.6)	0.323^b^
No	30 (88.2)	27 (79.4)	
Alcohol (*n*, %)			
Yes	0 (0)	0 (0)	
No	34 (100)	34 (100)	
IPAQ (*n*, %)			
Low	13 (38.2)	13 (38.2)	0.350^b^
Moderate	21 (61.8)	19 (55.99)	
High	0 (0)	2 (5.9)	

*Note: N*, number of participants; *X*, mean.

Abbreviations: BMI, body mass index; IPAQ, international physical activity questionnaire; SD, standard deviation; TTH, tension‐type headache.

^a^Independent samples t‐test.

^b^Chi‐square test.

Evaluation of headache characteristics in the TTH group demonstrated a mean symptom duration of 43.39 ± 37.15 months, with a frequency of 13.65 ± 8.22 headache days/month and average episode duration of 118.68 ± 169.86 min. Pain intensity scores on the NPRS were quantified as 6.23 ± 2.17 at rest, 7.07 ± 2.08 during activity, and 6.26 ± 2.91 at night. Of the 34 participants in the TTH group, 19 (55.9%) were classified as having episodic TTH and 15 (44.1%) as having chronic TTH, based on the ICHD‐3 frequency criteria. The headache characteristics of the TTH group are presented in Table 2.

**Table 2:** Headache characteristics of the tension‐type headache group.

Characteristics	TTH group (*N* = 34)		
	Mean ± SD	Lower	Upper
Pain onset time (month)	43.39 ± 37.15	31.1831	55.6064

Number of days with pain (day/month)	13.65 ± 8.22	10.9536	16.3622

Time of pain (minute)	118.68 ± 169.86	62.8501	174.5183

NPRS‐Rest	6.23 ± 2.17	5.5223	6.9514

NPRS‐Activity	7.07 ± 2.08	6.3938	7.7641

NPRS‐Sleep	6.26 ± 2.91	5.3066	7.2197

Medication use (*N*, %)	Yes	18 (52.9)	
	No	16 (47.1)	

Headache type (*N*, %)	Episodic	19 (55.9)	
	Chronic	15 (44.1)	

*Note: N*, number of participants; *X*, mean.

Abbreviations: NPRS, numeric pain rating scale; SD, standard deviation.

Cervical flexion (95% CI: −1.38 to −0.40, *d* = 0.90, *p* ≤ 0.001) and extension (95% CI: −2.02 to −0.97, *d* = 1.50, *p* ≤ 0.001) mobility and cervical flexor (95% CI: −1.49 to −0.49, *d* = 0.99, *p* ≤ 0.001) and deep neck flexor muscle endurance (craniocervical flexion test, 95% CI: −1.64 to −0.61, *d* = 1.13, *p* ≤ 0.001) scores were lower in women with TTH compared to the healthy controls, while cervical posture (craniovertebral angle, *p* = 0.638) and the cervical extensor muscle endurance (*p* = 0.144) scores were similar (Table 3).

**Table 3:** Spinal posture, mobility, and muscle endurance scores of the groups.

Spinal posture		TTH group (*N* = 34)	Control group (*N* = 34)	Difference between means (95% CI)	Effect size (d)	*p*
Cervical posture (craniovertebral angle, °, *X* ± SD)		50.50 ± 5.14	51.11 ± 5.78	−0.61 (−0.58, 0.35)	0.11	0.638^a^
Thoracic posture (sagittal angle, °, *X* ± SD)		40.05 ± 7.84	35.15 ± 8.42	4.9 (0.11, 1.08)	0.60	**0.015** ^**a**^
Lumbar posture (sagittal angle, °, *X* ± SD)		−36.65 ± 10.38	−26.96 ± 39.91	−9.68 (−0.81, 0.14)	0.33	0.170^a^
Sacral posture (sagittal angle, °, *X* ± SD)		19.00 ± 7.98	23.36 ± 28.22	−4.36 (−0.68, 0.26)	0.21	0.383^a^

Spinal mobility						

Cervical flexion mobility (range of motion, °, *X* ± SD)		50.10 ± 6.33	55.00 ± 4.23	−4.89 (−1.38, −0.40)	0.90	**< 0.001** ^**a**^
Cervical extension mobility (range of motion, °, *X* ± SD)		49.16 ± 7.44	58.76 ± 4.96	−9.6 (−2.02, −0.97)	1.50	**< 0.001** ^**a**^
Thoracic sagittal mobility (range of motion, °, *X* ± SD)		24.91 ± 11.00	24.24 ± 16.17	0.67 (−0.42, 0.52)	0.04	0.841^a^
Lumbar sagittal mobility (range of motion, °, *X* ± SD)		79.51 ± 11.83	78.66 ± 12.48	0.84 (−0.40, 0.54)	0.07	0.775^a^
Sacral sagittal mobility (range of motion, °, *X* ± SD)		44.80 ± 14.16	50.60 ± 14.04	−5.8 (−0.89, 0.07)	0.41	0.095^a^

Spinal muscle endurance						

Cervical muscle endurance tests	Flexion muscle endurance score (second, *X* ± SD)	14.43 ± 6.01	21.77 ± 8.62	−7.34 (−1.49, −0.49)	0.99	**< 0.001** ^**a**^
	Extension muscle endurance score (second, *X* ± SD)	43.22 ± 20.94	50.70 ± 21.44	−7.48 (−0.82, 0.12)	0.35	0.144^a^
	Craniocervical flexion test	25.29 ± 9.78	37.35 ± 11.47	−12.05 (−1.64, −0.61)	1.13	**< 0.001** ^**a**^

McGill trunk muscle endurance tests	Trunk flexion muscle endurance score (second, *X* ± SD)	15.64 ± 7.36	22.39 ± 8.57	−6.75 (−1.33, −0.35)	0.84	**0.001** ^**a**^
	Trunk extension muscle endurance score (second, *X* ± SD)	15.45 ± 10.19	17.66 ± 10.20	−2.21 (−0.68, 0.25)	0.21	0.368^a^
	Trunk right flexion muscle endurance score (second, *X* ± SD)	8.97 ± 3.41	14.97 ± 5.92	−5.99 (−1.75, −0.73)	1.24	**< 0.001** ^**a**^
	Trunk left flexion muscle endurance score (second, *X* ± SD)	9.43 ± 3.84	16.78 ± 7.40	−7.34 (−1.76, −0.73)	1.25	**< 0.001** ^**a**^

Sahrmann test	Level 1 (*n*. %)	12 (35.3)	5 (15.6)			**0.003** ^**b**^
	Level 2 (*n*. %)	20 (58.8)	14 (43.8)			
	Level 3 (*n*. %)	2 (5.9)	13 (40.6)			

*Note: N*, number of participants; °, degree; X, mean. The bold values indicate statistically significant results (*p* < 0.05).

Abbreviations: SD, standard deviation; TTH, tension‐type headache.

^a^Independent samples t‐test.

^b^Chi‐square test.

In addition, the thoracic angle (95% CI: 0.11 to 1.08, *d* = 0.60, *p* = 0.015) was higher in the sagittal plane, and the trunk flexor (95% CI: −1.33 to −0.35, *d* = 0.84, *p* = 0.001), trunk right/left lateral flexor muscle endurance (95% CI: −1.75 to −0.73, *d* = 1.24, *p* ≤ 0.001)/(95% CI: −1.76 to −0.73, *d* = 1.25, *p* ≤ 0.001), and trunk core stability (Sahrmann test, *p* = 0.003) scores were lower. However, the lumbar (*p* = 0.070) and sacral (*p* = 0.383) angles, the thoracic (*p* = 0.841), lumbar (*p* = 0.775), and sacral mobility (*p* = 0.095) in the sagittal plane, and the trunk extensor muscle endurance (*p* = 0.368) scores were similar (Table 3).

## 4. Discussion

The current study demonstrated that women with TTH exhibited significantly reduced cervical mobility in both flexion and extension, along with decreased endurance of the cervical flexor and deep neck flexor muscles compared to the healthy controls, while maintaining similar cervical posture and cervical extensor muscle endurance. Additionally, we observed an increased thoracic angle in the sagittal plane and reduced endurance of the trunk flexors and trunk right/left lateral flexors and trunk core stability in women with TTH compared to the healthy controls. However, the lumbar and sacral angles in the sagittal plane, as well as the thoracic, lumbar, and sacral mobility and the trunk extensor muscle endurance, were found to be similar between groups. Furthermore, a medium effect size was observed for thoracic posture, while large effect sizes were found for all other parameters.

Previous studies have evaluated cervical posture and mobility in individuals with TTH [28–30]. In a case‐control study conducted by Nagasawa et al., flattening of the cervical spine (assessed via X‐ray) was reported in individuals with TTH. The authors suggested that chronic contraction of head and neck flexor muscles may contribute to this cervical straightening [28]. Another case‐control study found that individuals with chronic TTH exhibited more pronounced forward head posture (measured by goniometer) and reduced cervical mobility compared to the controls [29]. A meta‐analysis by Liang et al. similarly reported more forward head posture and decreased cervical mobility in individuals with TTH [30]. Another meta‐analysis study showed that individuals with chronic TTH had more forward head posture compared to those with episodic TTH and healthy controls [7]. In our study, cervical posture was assessed using the craniovertebral angle (measured by goniometer), and similar results were found between groups. This discrepancy might be attributed to key methodological and sample characteristics. Firstly, the average headache frequency in our study (13.65 days/month) suggests a sample slightly in favor of episodic TTH, though very close to the chronic threshold. Additionally, our TTH group was a mixed sample consisting of 55.9% episodic and 44.1% chronic TTH participants. It is plausible that more pronounced postural adaptations, such as FHP, develop as a chronicity factor, potentially manifesting more clearly in individuals with chronic TTH (≥ 15 headache days/month) [31]. Secondly, the assessment method (goniometer vs. radiography) and the postural task (static standing vs. functional activity) can influence the findings [28]. Moreover, one should consider that primary headaches may not always be a sign of an underlying cervical musculoskeletal dysfunction [32, 33]. The lack of postural difference in our study, concurrent with significant deficits in mobility and muscle function, might indicate that impaired neuromuscular control precedes and potentially contributes to the development of observable static postural changes over time. Therefore, our results may reflect an earlier stage of dysfunction, where functional capacity is compromised before a static malalignment becomes evident. Our findings similarly showed lower cervical mobility in women with TTH.

Cervical muscle dysfunction is not the cause of TTH but rather a common comorbidity that may contribute to a worse manifestation of the disease, including increased frequency and intensity of headache episodes [34]. Impaired muscle function leading to reduced blood flow and metabolism has been associated with TTH [35]. In a case‐control study by Fernández‐de‐Las‐Peñas et al., individuals with chronic TTH showed lower endurance of the deep neck flexor muscles (craniocervical flexion test) compared to the healthy controls [36]. In adolescents with TTH, bilateral surface EMG data from the sternocleidomastoid muscle during isometric neck flexor endurance testing revealed significantly shorter total endurance time in TTH patients compared to the controls [35]. Another case‐control study by Del Blanco Muñiz et al. similarly found lower deep neck flexor endurance (craniocervical flexion test) in individuals with TTH compared to the healthy controls [37]. In contrast, a cross‐sectional study by Marcand et al. found similar neck extensor endurance between individuals with TTH and the controls [38]. Our results revealed a selective deficit in the endurance of cervical flexor muscles (both superficial and deep) while extensor endurance remained comparable to controls. This finding has substantial clinical implications. The deep neck flexors, in particular, are crucial for segmental stabilization and proprioception [36]. Their impairment can lead to poor neuromuscular control, altered load distribution across cervical segments, and increased strain on passive structures and more superficial muscles (e.g., the sternocleidomastoid and upper trapezius), which are common sites of tenderness in TTH [5]. The preserved extensor endurance might indicate a compensatory mechanism or a different adaptation pattern. This muscular imbalance—weakness anteriorly with maintained strength posteriorly—could potentially alter the cervical joint’s instantaneous axis of rotation, promote abnormal joint loading, and contribute to the perpetuation of nociception. This selective deficit underscores the importance of specifically assessing and treating flexor endurance in the rehabilitation of individuals with TTH, rather than prescribing general strengthening exercises.

According to the motor adaptation to pain theory, protective postural changes can occur even in regions distant from the pain site [9]. Additionally, the lower physical activity levels commonly observed in individuals with chronic migraine may lead to impairments in general posture, mobility, and muscle endurance functions [39]. Indeed, a case‐control study by Ozudogru Celik et al. in migraine patients showed higher thoracic angle (measured by the DIERS Formetric 4D motion imaging system) compared to the healthy controls [40]. Another study by Moustafa et al. on cervicogenic headache reported higher thoracic and lumbar angles and spinal rotation (measured by the DIERS Formetric 4D motion imaging system) in individuals with chronic cervicogenic headache compared to the controls [41]. In a different study by La Touche et al. on migraine patients, trunk muscle endurance was found to be lower compared to the healthy controls [39]. However, we identified only one previous study examining thoracic, lumbar, and sacral posture in individuals with TTH. That study evaluated lumbopelvic angle (using biophotogrammetric evaluation) and found it to be higher in individuals with chronic TTH compared to the healthy controls [12]. No previous studies were found examining thoracic, lumbar, and sacral mobility or trunk muscle endurance in TTH patients. In our study, we observed a greater thoracic angle in sagittal plane and lower trunk flexor and right/left lateral flexor muscle endurance and trunk core stability in women with TTH. However, lumbar and sacral angles in sagittal plane, as well as thoracic, lumbar, and sacral mobility and trunk extensor muscle endurance, were similar between groups. Adopting a whole‐spine perspective, our findings of an increased thoracic kyphosis angle without concomitant changes in lumbar or sacral posture or mobility in any region beyond the cervical spine offer a nuanced view of motor adaptation in TTH. Our results suggest that this adaptation in TTH is not a global spinal phenomenon but rather a focused one, primarily affecting the cervicothoracic region. The increased thoracic kyphosis could be a compensatory strategy to minimize mechanical stress on the painful cervical structures by reducing the demand on cervical stabilizers and altering the line of gravity. The lack of change in lumbar and sacral kinematics might indicate that the compensatory mechanism is efficiently contained within the upper quadrant or that our sample, with episodic TTH, had not yet developed downstream adaptations. This finding highlights that the primary dysfunctional unit in TTH might be the “cervicothoracic junction” rather than the cervical spine in isolation. Rehabilitation approaches, therefore, should not only target the cervical spine but also assess and address impairments in thoracic mobility and posture to effectively modify this potentially maladaptive pattern.

Another aspect worthy of discussion is the considerable variability observed in some of our outcome measures. The large standard deviation in headache episode duration (approximately 170 min) suggests substantial heterogeneity in pain experiences within our TTH study, which is a common characteristic of headache disorders and may reflect variations in pain perception, coping strategies, or headache subtypes. Similarly, the high variability in the lumbar posture of the control group (SD≈40°) could be indicative of a wide range of normal sagittal alignment in asymptomatic young women. This variability might also be influenced by the sensitivity of the Spinal Mouse device to subtle alterations in standing posture during assessment. While these variations enrich the generalizability of our findings by representing a more realistic population spectrum, they also highlight the importance of interpreting posture and headache characteristics on an individual basis in clinical practice, rather than relying solely on group averages.

### 4.1. Limitations of the Study

To our knowledge, this is the first study to evaluate thoracic, lumbar, and sacral posture and mobility, along with trunk muscle endurance, in women with TTH in addition to cervical assessment. However, this study has several limitations. The first limitation is the heterogeneity of our TTH sample. We included individuals with a general diagnosis of TTH without distinguishing between episodic and chronic subtypes. This methodological choice was made to initially investigate the potential presence of spinal alterations in a broader TTH population, as the primary aim of our study was to compare women with and without TTH. However, we acknowledge that this is an important limitation, as episodic and chronic TTH may involve different pathophysiological mechanisms and degrees of central sensitization, which could lead to divergent findings in spinal posture, mobility, and muscle endurance. Indeed, the average headache frequency in our sample was 13.65 days/month, which is near the chronic threshold but still reflects a slight predominance of episodic TTH. Our sample consisted of a mixed population (55.9% episodic and 44.1% chronic). This mixed composition may explain why we did not detect significant differences in some measures, such as cervical posture, compared to studies that included only chronic TTH patients. Future case‐control studies specifically designed with adequate power to compare these subgroups are needed to elucidate the potential differences between episodic and chronic TTH. Second, this study was conducted at a single center, and results might vary in patients from different regions. Third, the use of self‐reported headache characteristics without diary‐based verification is a limitation of this study, as it is susceptible to recall bias. Furthermore, the recruitment of control participants from patients’ relatives, while practical, may introduce selection bias. Fourth, although participants receiving physiotherapy or interventional treatment were excluded, the potential influence of concurrent medication use (e.g., analgesics and muscle relaxants) on posture, mobility, and muscle endurance was not controlled for. This is an important limitation that may affect the internal validity of our findings. Future studies should consider documenting and statistically controlling for medication use or implementing washout periods where it is ethically feasible to better isolate the relationship between TTH and spinal biomechanics. Fifth, although potential confounding factors such as BMI and physical activity level were collected and showed no significant differences between groups at baseline, they were not adjusted for in the statistical analyses. While group equivalence minimizes some concern, the use of analysis of covariance (ANCOVA) or multivariate regression models could have provided a more robust adjustment for these variables and strengthened the internal validity of our findings. Future studies with larger sample sizes should employ such statistical techniques to control for potential confounders and better isolate the specific effect of TTH on spinal biomechanics. Finally, the lack of correlation analyses between headache characteristics (intensity, frequency, duration) and the observed spinal parameters is another limitation. Such analyses could provide valuable insights into how spinal dysfunction relates to the clinical presentation of TTH and are recommended for future studies. Furthermore, we did not control for the presence or intensity of headache pain during the assessment sessions. Although participants were not restricted from using their prescribed analgesics, the potential for acute headache pain to influence performance on mobility and endurance tests must be considered a limitation, as it may have contributed to the observed differences between groups.

## 5. Conclusion

The findings of this study demonstrate that women with TTH exhibit significantly reduced cervical mobility and lower cervical muscle endurance compared to healthy women, while maintaining similar cervical posture. Furthermore, the results reveal that women with TTH show an increased thoracic kyphosis angle in the sagittal plane and decreased trunk muscle endurance relative to their healthy counterparts.

These observations suggest that comprehensive evaluation and management of TTH in women should incorporate assessment of the specific spinal impairments identified, namely, thoracic posture in the sagittal plane, spinal mobility, and muscle endurance, as potentially important components of therapeutic interventions. Although cervical posture was similar between groups, the alterations in thoracic alignment, alongside the deficits in mobility and muscle endurance, highlight the need for a broader spinal evaluation beyond the cervical region.

Future research should aim to validate these findings across diverse population groups, including investigations in male subjects and individuals with different headache subtypes. Such studies would contribute to a more comprehensive understanding of the biomechanical factors associated with TTH and facilitate the development of more effective, individualized treatment strategies.

## Ethics Statement

The study was conducted in accordance with the Declaration of Helsinki and Good Clinical Practice guidelines. The present study protocol was reviewed and approved by the institutional review board of Bitlis Eren University (Approval No. 2024/3‐2, Date: 04/04/2024). Informed consent was submitted by all subjects when they were enrolled.

## Conflicts of Interest

The authors declare no conflicts of interest.

## Author Contributions

Mesut Arslan and Sonay Guruhan: conceptualization, data curation, formal analysis, funding acquisition, investigation, methodology, project administration, resources, writing–original draft, and writing–review and editing. Seyda Toprak Celenay: conceptualization, formal analysis, funding acquisition, investigation, methodology, project administration, resources, and writing–review and editing.

## Funding

Wiley and Türkiye Bilimsel ve Teknolojik Araştırma Kurumu have an agreement which allows eligible authors to publish open access without paying an article publication charge (APC). The cost of publishing is covered under the terms of the agreement.

## Data Availability

The data that support the findings of this study are available on request from the corresponding author. The data are not publicly available due to privacy or ethical restrictions.
